# Synergies and dis-synergies between universal health coverage and global health security: A case study of Cambodia

**DOI:** 10.7189/jogh.14.04218

**Published:** 2024-11-15

**Authors:** Lo Yan Esabelle Yam, Pheak Chhoun, Di Liang, Jiayan Huang, Siyan Yi

**Affiliations:** 1 Saw Swee Hock School of Public Health, National University of Singapore and National University Health System, Singapore, Singapore; 2 College of Health and Medicine, Australian National University, Canberra, Australia; 3 KHANA Center for Population Health Research, Phnom Penh, Cambodia; 4 Department of Hospital Management, School of Public Health, Fudan University, Shanghai, China; 5 Public Health Program, College of Education and Health Sciences, Touro University California, Vallejo, California, USA

## Abstract

**Background:**

After the coronavirus disease 2019 (COVID-19) pandemic, the global community’s increased focus on pandemic preparedness has driven efforts such as the Pandemic Treaty proposed by the World Health Organization (WHO) and the Pandemic Fund managed by the World Bank. While these initiatives will enhance countries’ capabilities in pandemic preparedness, synergies could be achieved by exploring the intersections between universal health coverage (UHC) and global health security (GHS). This is particularly relevant for developing countries like Cambodia. As it transitions to higher income status and reduces its reliance on external funding, the synergistic development of UHC and GHS will help Cambodia maximise health investment and align with its population health goals. We aimed to identify synergies and dis-synergies between UHC and GHS and recommend implementations that the government can consider moving forward.

**Methods:**

We conducted a rapid review of policy documents based on the World Health Organization (WHO) Health System Framework and undertook consultations with key stakeholders in Cambodia.

**Results:**

Our findings show the synergies between the two agendas in Cambodia resulted from having a central coordinating authority through the Ministry of Health (MoH), an extensive primary care network, and intersecting human resources that drive both UHC and GHS. We also identified potential dis-synergies such as vertical programmes and funding sources, inadequate regulation and engagement of the private sector, and underutilisation of information and data. Recommendations include cross-consultations between departments within the MoH when developing policies in GHC or UHC, and training programmes to increase awareness of the synergies between UHC and GHS.

**Conclusions:**

Our findings reinforce those of previous case studies in Bangladesh, Ethiopia, and Ghana, offering recommendations for building resilient health systems by integrating UHC and GHS.

Over the past decades, Cambodia, a lower-middle-income country in Southeast Asia, has significantly improved its population health, with increases in its universal health coverage (UHC) service coverage index [1]. However, parallel to this upward trend, the country’s out-of-pocket expenditure per capita has also been rising since 2007 [2]. While routine healthcare services were largely unaffected during the coronavirus disease 2019 (COVID-19) pandemic [3], movement restrictions and fears of infection impacted Cambodians' healthcare service utilisation [4]. The UHC service coverage index took a slight dip in 2019 and has remained at the same level in 2021 [1]. The economic impact of the pandemic has also resulted in income loss and unemployment, with approximately 460 000 people falling into poverty in 2020 [5]. Although social assistance was given to 700 000 low-income households, there were still concerns about the adequacy of cash support and effectiveness in reaching the unregistered poor [6,7].

The impact of the pandemic on the country's progress towards UHC and economic growth underscores the importance of investing in the capacity and capability to manage health security threats. As the global community continues to focus on pandemic preparedness through efforts such as the Pandemic Treaty [8], the Pandemic Fund [9], and pandemic preparedness plans [10], advocates have called for the alignment, synergies, and integration of global health security (GHS) with UHC to build resilient health systems [11–14]. The argument is that effective management of infectious diseases and outbreaks requires robust health systems capable of providing quality essential health services with financial protection, which, in turn, builds trust and improves population health, better preparing the country to cope with threats of communicable diseases.

The conceptual understanding of the intersections and tensions between UHC and GHS has garnered much debate and interest over the years [15]. However, a few studies attempted to understand the intersections at the country level [9,16–18]. As Cambodia looks back at the lessons learned from the pandemic, moves forward in strengthening its health system for pandemic preparedness, and progresses toward UHC, this window provides an opportunity to assess past developments of UHC and GHS in its context to inform plans moving forward. We aimed to assess the policies, implementations, and practices in UHC and GHS in Cambodia to identify areas for synergies and dis-synergies between the two agendas and recommend implementation approaches that the government can consider moving forward.

## METHODS

We conducted a rapid review in April 2023 to gain an overview of the policy context on UHC and GHS in Cambodia. We followed this up with a consultative meeting with key stakeholders from governmental and non-governmental organisations (NGOs) to discuss the synergies and dis-synergies between the two agendas.

For the rapid review, we designed a search strategy based on terms related to UHC and GHS, connected by Boolean operators where appropriate (**Table 1**), to identify documents issued by the Government of Cambodia through Google Scholar and the Cambodian government’s websites. We set no specific dates for our search. We also obtained documents from government stakeholders present at the above-mentioned consultative meeting. We included any government policies, plans, strategy documents, presentation slides, and legal documents containing health security or UHC in our analysis. We reviewed documents in English and Khmer; when a document was available only in Khmer, we asked a local Cambodian researcher to translate the relevant sections.

**Table 1:** Search terms for the rapid review*

Global health security	Universal health coverage
Global (health security)	Universal health coverage
(Pandemic) preparedness	(Health equity) fund
Outbreak management/response	IDPoor
Emergency management/response	Social health protection
	Social protection
	Health insurance

*Words in brackets are search terms that form another search term when added to the words outside the bracket.

We then organised the data using the World Health Organization (WHO) Health System Framework: leadership, service delivery, health financing, health workforce, health information systems, and medical products and technologies [19].

The consultative meeting was organised to introduce and discuss the concepts of synergies and dis-synergies between UHC and GHS with representatives from the Cambodian Ministry of Health (MoH), Ministry of Labour and Vocational Training, Ministry of Economy and Finance, and NGOs. In this process, we used the WHO’s definitions of ‘UHC’ and ‘health security’ and described the term ‘synergies’ as resulting in a substantial positive effect on achieving desired outcomes in UHC and GHS agendas [20–22]. Conversely, ‘dis-synergies’ result in substantial adverse effects. We shared a draft report containing the review and insights from the meeting with the meeting participants and used their feedback to refine the content presented here.

## RESULTS

### Country and health system developments

Following civil unrest in the 1970s and 1980s, the government focussed its domestic policies on peace, security, and socioeconomic development. The national constitution, adopted in 1993, Article 72, states: ‘The health of the people shall be guaranteed. The State shall give full consideration to disease prevention and medical treatment. Poor citizens shall receive free medical consultation in public hospitals, infirmaries, and maternities. The State shall establish infirmaries and maternities in rural areas’ [23]. These declarations underpin the country’s commitment to UHC and the recognition of health for all. By framing this within the context of nation-building, it acknowledges the interdependency of access to health with social welfare and national security.

In the post-conflict recovery period, the government’s lack of self-sufficiency necessitated heavy reliance on international organisations to drive its development. In health, the focus was predominantly on vertical communicable disease programmes, notably HIV, tuberculosis (TB), malaria, and reproductive, maternal, newborn, and child health. Aid delivery was fragmented among the international actors, and there was a preference for vertical programmes over system-level investments [24]. This approach limited synergies between UHC and GHS.

Nonetheless, beginning in the 1990s, the government introduced health reforms to coordinate sector-wide planning and financing of health services. A significant reform was the introduction of the sector-wide approach (SWAp), which facilitated the collaboration of development partners within a unified framework of national goals, strategies, and budgets [25]. This approach supported the national objectives outlined in the Health Strategic Plans (HSPs). The HSPs for 2003–2007 [26], 2008–2015 [27], 2016–2020 [28], and 2022–2030 [29] shared the overarching goal of achieving UHC, expanding health access and coverage, and ensuring financial risk protection. Notably, the 2022–2030 HSP has extended its focus on UHC to healthcare across the life course, from health promotion, prevention, and curative to rehabilitation and palliative care, in alignment with the WHO’s definition of UHC [20].

The HSP 2022–2030 identified health security threats beyond outbreaks to antimicrobial resistance (AMR), climate change, environmental health risks, and disaster risk management [29]. These priority areas underscore the necessity of enhancing core capacities and capabilities to meet the International Health Regulations’ requirements and strengthening preparedness and response to health security threats beyond communicable diseases.

The HSP 2022–2030 also emphasises a health system approach, which includes strengthening health financing infrastructure for UHC, equipping primary health centres to respond to communicable diseases and public health threats effectively, and broadening local capacity for preparedness and response through community engagement in health promotion and preventive care [29]. This approach transcends the dichotomous UHC vs GHS narrative [30]. Overall, the strategic emphasis on GHS in support of the broader goal to advance UHC is rooted in system-level enhancements that foster synergies between the two agendas.

### Leadership

**Figure 1:**
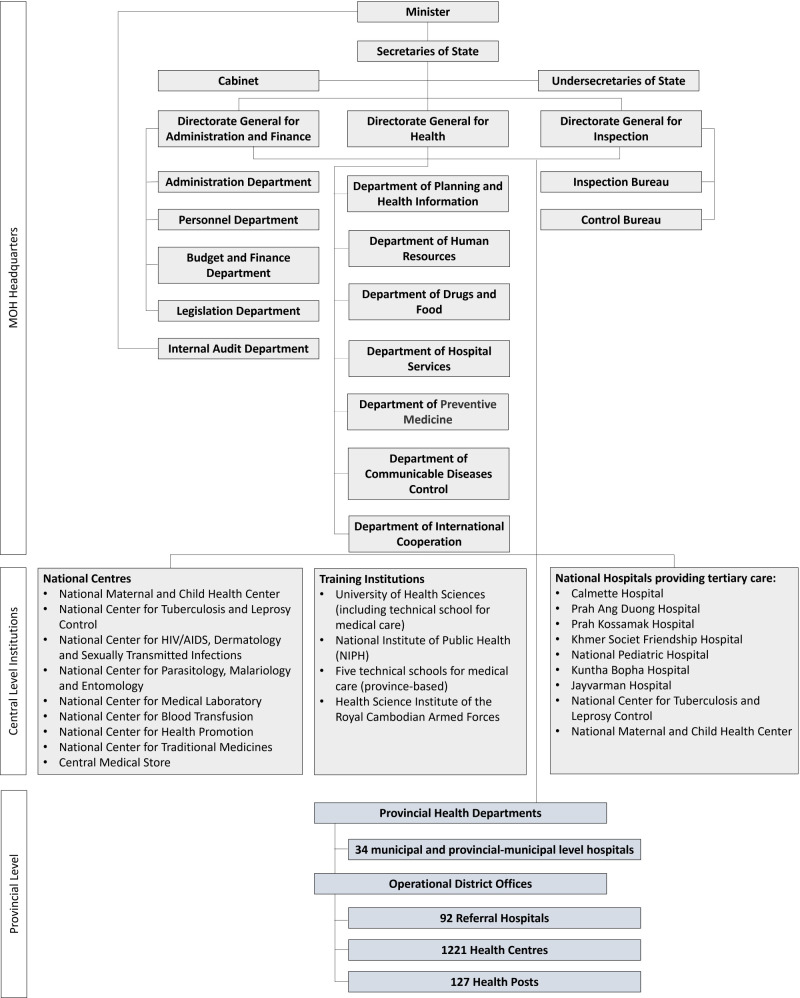
Organisation structure of MoH and public health facilities in Cambodia [25,31,32].

The MoH collaborates within and with other ministries to advance UHC and GHS (**Figure 1**) [25]. In 2019, the Government of Cambodia delegated health management and service provision to 25 municipal and provincial administrations [32]. On financial protection, the MoH works closely with the Ministry of Planning in managing the Health Equity Fund, which provides financial coverage for essential health services to the poor. The MoH is also part of the National Social Protection Council, established in 2017 to harmonise government-wide social security and social assistance schemes.

Health security activities are primarily coordinated by MoH’s Department of Communicable Disease Control, the focal point for implementing the International Health Regulations, in collaboration with other MoH departments and national centres [25]. The MoH’s central role in coordinating UHC and GHS could be leveraged to foster synergies. However, the concept of synergies was not intuitive to participants at the consultative meeting. For the effective integration of UHC and GHS, the concepts of synergies between the two agendas would need to be discussed within the country's specific context to make them relatable to the stakeholders driving implementations for both agendas.

### Health service delivery

A robust primary healthcare system is the bedrock of UHC, enabling people to access essential health services whenever and wherever required. Additionally, it supports GHS by facilitating the surveillance, prevention, and control of communicable diseases. In Cambodia, a network of health centres provides a range of primary health services, including surveillance and management of communicable diseases, complemented by para-medical support such as laboratory services and infection prevention and control. However, suboptimal quality and low utilisation of public health services have been reported [33,34]. To address the demand side of healthcare utilisation, it is vital to increase the community's awareness of the services available in public facilities and promote trust in using the services through quality improvement.

Parallel to the public services are over 8000 private health facilities, which are patients’ preferred choice [28,35]. The private health sector operates with minimal government regulation, leading to a spectrum of services and variable service quality. This poses a challenge in achieving UHC, particularly in ensuring equitable access to quality healthcare for all. Inadequate regulation also creates a gap in disease surveillance. Although private health providers are required to submit reports to the government, their interactions with the government predominantly revolve around licensing and administrative matters rather than outbreak management and population health reporting [36].

### Health financing

In 2020, out-of-pocket expenditure constituted 61%, government expenditure 28%, and external health expenditure 7% of the total health expenditure, while the remainder went towards social health insurance and voluntary health insurance [2]. A significant portion of out-of-pocket expenditure was incurred in private health facilities [37]. External health expenditure from development agencies primarily targeted HIV and AIDS, followed by reproductive, maternal, newborn, and child health, malaria, and TB [37]. While the government has worked to consolidate external funding through reforms such as SWAp, some continue to operate in tandem with its system, potentially contributing to dis-synergies between UHC and GHS if not effectively aligned with broader national health priorities.

Government expenditure supports public health and healthcare infrastructure, staff, and subsidised care. Budget allocations are based on priority areas outlined in the HSP. Funded by external and government agencies, TB testing and treatment, HIV testing, antiretroviral therapy, and expanded immunisation programmes are provided free of charge at public facilities. These free services help prevent and control communicable diseases, bolster surveillance efforts, and foster progress toward UHC.

Social health insurance, such as the Health Equity Fund managed by the MoH, offers financial coverage for essential health services to individuals listed in the IDPoor registry and certain informal workers. As of 2017, 16% of the population has been covered by the Health Equity Fund [31], which is then complemented by the social and health insurance scheme for non-poor citizens managed by the National Social Security Fund (NSSF) under the Ministry of Labor and Vocational Training and the Ministry of Economy and Finance. According to findings from the consultative meetings, NSSF covers healthcare services for non-work and work-related injuries through 13 000 public and 125 private health facilities and currently serves three million individuals from the private and public sectors, managing social insurance schemes encompassing work injuries, sickness, maternity, and pension schemes. Informal workers are eligible for NSSF membership, and efforts are under way to expand the coverage to family members of NSSF cardholders by the end of 2023, potentially including an additional three million individuals.

HEF and NSSF’s coverage enhances access to health services with financial protection, contributing to individual-level security and promoting collective security at community and national levels [38]. Furthermore, by addressing the demand-side barriers to care through financial protection and supply-side improvements in healthcare quality, these schemes support health-seeking behaviours that can support both agendas. One key concern is the low utilisation of HEF among its members, which needs to be addressed by increasing awareness of its benefits and access.

### Health workforce

Cambodia faces a shortage of medical professionals, and dual practice in the public and private sectors is common [39,40]. Supporting facility-based health workers are community health workers, such as the village health support groups, who are crucial in connecting health centres with the communities. They disseminate health messages, conduct outreach activities, and provide essential health education within the communities.

At the health centre level, HIV and AIDS, TB, and malaria programmes are overseen by trained secondary or primary nurses. Although most staff lack laboratory training, they can perform basic ancillary tasks such as TB and malaria tests, which are then subsequently confirmed at referral hospitals. The health centre management committees foster community engagement in health services and organise education campaigns for infectious disease prevention. The health centres also collaborate with MoH’s Department of Communicable Disease Control, which has a rapid response team comprising over 2000 members from provincial health departments and operational districts, in outbreak management [6].

Specialised working groups in infection control are established across hospital departments at the referral hospitals. These groups facilitate infection control measures and coordinate efforts during disease outbreaks. Quality improvement working groups are tasked with implementing quality improvement policies and guidelines, a pivotal element in enhancing service quality. The membership of these working groups often overlaps, enabling effective communication at operational levels in areas that intersect with both agendas.

The National Institute of Public Health has conducted leadership and management training for managers and senior staff at the health centres. This initiative enhances competencies across analytics and assessment, communication, programme planning, community engagement, financial planning and management, leadership, and systems thinking, as well as emergency preparedness, planning, and response [41]. Similar training initiatives are planned for management staff at the referral hospitals. Such training is essential for bolstering the capabilities of health centres to deliver quality health services and manage emergencies effectively.

Most continuing professional education was funded by international development, NGOs, and private organisations [25]. The training programmes focus on disease-specific activities which inadvertently limit the synergies between UHC and GHS. Continuing professional education should incorporate interlinkages between agendas to enable healthcare workers to appreciate their work beyond their programmes or immediate responsibilities. This approach would support them in championing synergistic initiatives between UHC and GHS in their day-to-day operations.

### Health information systems

The MoH launched its first Health Management Information System in 1993 [25], which is now complemented by the Health Equity Fund Operational Database, known as the Patient Medical Registration System, overseen by the Health Management Information System’s Bureau within the MoH’s Department of Planning and Health Information. The MoH has also introduced specialised information systems to address specific health challenges such as HIV and AIDS, TB, malaria, non-communicable diseases, drug inventory management, health financing, and national surveys.

The Health Information System Master Plan 2016–2020 recognised the use of health information in designing the expansion of health services and identifying emerging public health threats [42]. It also highlighted challenges such as data quality and accuracy. For communicable disease surveillance, the MoH’s Department of Communicable Disease Control uses a national surveillance system called the Cambodia Early Warning System, which collects and aggregates data on 10 epidemic-prone diseases in Cambodia [43]. Its goal is to monitor disease trends and detect outbreaks early to facilitate a timely response by the rapid response teams. However, the surveillance infrastructure remains fragmented. Sub-national systems employ indicator-based surveillance at the health centre level, while the national level utilises the Cambodia Early Warning System’s database, an event-based surveillance system facilitated through hotlines for real-time reporting by health facilities.

The lack of clear data governance has limited the enforcement of Patient Medical Registration System usage in private health facilities. Furthermore, the lack of integration between health information systems in the public and private sectors hampers the linkage of data sets for understanding healthcare utilisation patterns, disease prevalence, and evolving health demands. This fragmentation also leads to inefficiencies in resource allocation and impairs the ability to promptly detect emerging or re-emerging communicable diseases and other health threats, while also limiting the use of data to fulfil the synergistic potential between developments in UHC and GHS.

### Medical products and technologies

The availability of essential drugs, diagnostic tests, and vaccines is a key component supporting UHC and GHS. The Health Product Strategic Plan 2023–2030 aims to ensure access to medicines of assured quality, safety, and effectiveness for all Cambodians by improving oversight of medical products through enhanced adherence to legal standards, investing in human resources to effectively perform market authorisation and registration, strengthening supply chain structures to counter substandard and falsified medical products, and developing a monitoring framework to ensure efficient use of health products at public health facilities [44].

Cambodia’s population often obtains medicines from private pharmacies and drug stores as their first point of contact with the health system [45]. The presence of counterfeit medicines and unregulated pricing and prescribing contribute to out-of-pocket expenditure and AMR [46], consequently corroding the trust in the healthcare system and affects health-seeking behaviour, potentially compromising health outcomes [46,47]. In the context of a health emergency, an unregulated drug market can lead to the use of unverified or unregulated drugs, price gouging, and inequitable medication access, thereby hindering the government’s ability to provide appropriate treatment options and control disease spread [48]. Disruptions to the supply chain, as shown during the pandemic, can affect the availability of health products [49]. In Cambodia, where most drugs and vaccines are imported and procured through the United Nations Children’s Fund and the private sector, strengthening the supply chain is critical for the health system in handling health emergencies and providing essential health services.

## DISCUSSION

Cambodia has set its sights on advancing toward UHC, as outlined in its constitution and the national strategic plans, by bolstering health security through a system-wide approach. The country’s focus on health security threats has evolved over time; it began with vertical programmes addressing HIV, TB, and malaria, but has since expanded to encompass zoonotic diseases, AMR, climate change, environmental health risks, and pandemic preparedness. The synergies between UHC and GHS within the healthcare system could be fostered by leveraging MoH’s central coordinating role, the extensive network of health centres, and intersecting memberships of technical working groups on infection control and quality improvement.

Nevertheless, dis-synergies could arise from vertical disease programmes, funding from international donors alongside government funding, inadequate regulation and the exclusion of private healthcare providers from outbreak management, and fragmented information systems. These challenges are not unique to Cambodia. For example, in Ghana, where efforts using the SWAp to consolidate donor funds eventually reverted to selective programme support, causing potential fragmentation in financing the health system [17]. In Ethiopia, different reporting formats to various funders have led to fragmentation. Recognising this issue, the Ethiopian government launched an Information Revolution Roadmap to coordinate donors and implementing partners working on its Health Management Information System towards making the system more unified [18]. Notably, the concept of synergies was not intuitive to participants at the consultative meeting in Cambodia. This mirrors findings from the study in Bangladesh, which reported little conceptual clarity among participants about the interlinkages between the agendas [16].

These dis-synergies and challenges can hinder the Cambodian government’s efforts to advance UHC and GHS. To address the need for a better understanding of interlinkages, the National Institute of Public Health and universities should consider including the concepts of GHC and UHC and their synergies in their training programmes. The Government of Cambodia, meanwhile, could enhance local capabilities through knowledge transfer from development partners to address gaps in the health system, such as a weak domestic procurement and supply chain. It could also more deeply involve the private sector in supporting disease surveillance, treatment, and control by exploring public-private partnership models from other countries and using legal or regulatory instruments [50]. The government could also explore ways to further support the existing synergies, for example by ensuring policy development is driven by broad consultations across the MoH’s departments. For example, on matters relating to GHS, such as pandemic preparedness, the views of those responsible for social health protection should be included.

The COVID-19 pandemic has underscored the importance of securing health security in enabling the country to make progress towards UHC by mitigating the impact of health crises that can impede the population’s access to healthcare. Conversely, progress to UHC can contribute to a healthier population that responds better during pandemics. Therefore, national and global stakeholders involved in shaping and investing in Cambodia’s health system must recognise the value of synergies between these two development agendas in building a resilient and responsive health system. As Cambodia transitions to a higher-income country status and receives reduced external funding, health programmes should be designed to be synergistic to maximise the value of investment. The focus should also be geared towards strengthening primary healthcare and moving care upstream to prevention and promotion to improve population health.

This study adds value by exploring the synergies and dis-synergies between UHC and GHS in Cambodia, offering a practical illustration of the two agendas in practice. It also reinforces findings from case studies in Bangladesh, Ethiopia, and Ghana, while simultaneously highlighting potential areas that the Cambodian government can consider in increasing synergies between the two agendas to build a resilient health system. Our own findings were further affirmed by the stakeholders themselves, as we have circulated a draft report of the review and solicited their verification and feedback after the meeting. A major limitation of our approach, however, is that we primarily relied on documents in English and only translated certain sections of documents in Khmer. Furthermore, we based our review only on government documents available in public domains and those provided by government representatives, possibly resulting in the omission of key documents. 

## CONCLUSIONS

The COVID-19 pandemic has shown that health security can only be achieved when essential health services are available and when access to health services is not disrupted by security threats. In a resource-constrained country like Cambodia, it is vital to be deliberate in identifying and achieving synergies between UHC and GHS. Through this study, we identified synergies between the two agendas in Cambodia that resulted from having a central coordinating authority, an extensive primary care network, and intersecting human resources that drive UHC and GHS. We also identified potential dis-synergies such as vertical programmes and funding sources, inadequate regulation and engagement of the private sector, and underutilisation of information and data. As Cambodia and countries worldwide work to strengthen GHS, they must not lose sight of the overarching goal of ensuring UHC for all.
